# Schizophrenia Patients With Prevotella-Enterotype Have a Higher Risk of Obesity

**DOI:** 10.3389/fpsyt.2022.864951

**Published:** 2022-05-30

**Authors:** Ying Liang, Yang Shen, Gaofei Li, Ye Yuan, Meng Zhang, Jiayu Gao

**Affiliations:** ^1^National Clinical Research Center for Mental Disorders, Peking University Sixth Hospital, Key Laboratory of Mental Health, Ministry of Health, Institute of Mental Health, Peking University, Beijing, China; ^2^Department of Psychiatry, Beijing Hospital of Integrated Traditional Chinese and Western Medicine, Beijing, China; ^3^Beijing Gene Tangram Technology Co., Ltd., Beijing, China; ^4^School of Chemical Engineering and Pharmaceutics, Henan University of Science and Technology, Luoyang, China

**Keywords:** schizophrenia, 16S rRNA sequences, gut microbiota, enterotypes, obesity

## Abstract

Recent studies have indicated the critical influence of gut microbiota on the occurrence of obesity. There is a significant risk of obesity in people with schizophrenia. This work proposed that the disorder of gut microbiota in patients with schizophrenia was based on microbial enterotypes. Ninety-seven patients with schizophrenia and 69 matched health controls were eligible. The fresh feces of all the subjects were collected and used to complete 16S rRNA sequence. Statistical analysis was performed to identify the intestinal type of gut microbiota and analyze their potential effects on metabolic function. The patients with enterotype-P had a higher BMI than that of the others. Several differences in the gut microbes of enterotype-P were found between the patients and the controls. Proteobacteria and Firmicutes had significantly higher abundance in the patients’ group with enterotype-P. The Bacteroidetes had higher abundance in health controls with enterotype-P. Different metabolic pathways of the microbiota with the enterotype-P were identified in the subjects categorized in different BMI intervals. The schizophrenia patients had a significantly higher BMI than that of health controls. The patients with enterotype-P had a higher BMI. Therefore, the enterotype-P might have a critical influence on a variety of metabolic pathways to disturb the metabolism of glucose and lipid in human body.

## Introduction

Patients with schizophrenia have been known to be at higher risk to suffer metabolic disorders. The meta-analysis study indicated that patients with schizophrenia had complications, such as obesity, abnormal blood glucose, and lipids, and even metabolic syndrome, before receiving antipsychotic medication treatment (1). Moreover, treatment with antipsychotics, especially second-generation antipsychotics, could further aggravate metabolic abnormalities. The previous research reported that the drug-naïve first-episode patients might experience significant weight gain and even develop obesity within 1 month under the antipsychotics treatment (2). These factors could adversely affect the compliance of the patients and the occurrence and development of physical diseases, which caused a significantly shorter life span (nearly 25 years) of the patients with schizophrenia than that of their peers (3, 4).

In addition, obesity could also aggravate brain atrophy (5). A schizophrenia study claimed that obesity could be an independent factor affecting the brain health of patients with first-episode schizophrenia, and the aging of the brain appeared earlier than the course of schizophrenia (6). The results suggested that obesity could be one of the factors that promote and sustain structural changes in the brain of patients with schizophrenia, thus ultimately contributing to the cognitive decline (7). Therefore, psychiatrists need to be proactive about metabolic issues, especially obesity in schizophrenics (8).

A number of studies have shown that obese people have the dysbiosis of gut microbiota. The increases of the permeability of the mucosal barrier could allow the bacteria and their products to enter the circulatory system to stimulate an inflammatory response (9). Meanwhile, visceral fat could further promote cellular aging and inflammation. The underlying mechanisms may include genomic instability, cell senescence, mitochondria dysfunction, microbiota composition changes, NLRP3 inflammasome activation, etc (10). These mechanisms could be associated with the development of schizophrenia as well. For example, patients with schizophrenia have low-level inflammation, different degrees of mitochondrial function damage, gut microbiota disorder, and so on (11). Therefore, schizophrenia and obesity may have a common pathogenic mechanism through the dysbiosis of gut microbiota.

In order to clarify the relationship between classification and function of gut microbiota, Manimozhiyan Arumugam et al. firstly proposed that human intestinal microorganisms could be divided into three types of enterotype, including enterotype-Bacteroides (B), enterotype-Prevotella (P), and enterotype-Ruminococcus (R) (12). Among them, the normal population with enterotype-P had a slightly higher BMI than those of the population with other enterotypes. The formation of these enterotypes has no obvious relationship with the age, sex, cultural background, and geographic location of the population but could be affected by long-term diet habits (13, 14). Another study also found a potential link between metabolic syndrome with diet and enterotype. Therefore, this study hypothesized that obese schizophrenics have the altered microbial enterotypes that may increase BMI.

## Materials and Methods

### Subjects

All the subjects in this study were required to sign an informed consent form. According to the Helsinki Declaration, the sample collection and data analysis were performed with the approval of the Ethics Committee of Kangning Hospital in Liaoning Province.

All the subjects are the Han nationality and live in Huludao, Liaoning Province, China. They have no special religious beliefs or diet habits. Ninety-seven patients with schizophrenia and 69 healthy controls were qualified and recruited. All the subjects were aged between 18 and 65 years; BMI was between 18 and 35 kg/m^2^, and their weight was stable without significant change in the past 3 months. The members of the patient group were diagnosed as schizophrenia according to the SCID- IV-TR diagnostic manual, and were not accompanied by other types of mental disorders, personality disorders, and intellectual disability. All the patients were in the maintenance phase, with negative symptoms predominately and no observable positive symptoms; the subjects of the control group were free of any mental disorders, personality disorders, or intellectual disability. According to WHO standards (15), 18.5 ≤ BMI < 25 is considered as normal; BMI ≥25 is considered as overweight or obesity. All the patients received a steady dose of antipsychotic drugs, such as risperidone, quetiapine, chlorpromazine, etc.

Based on our previous research (16), the patients with the following conditions were excluded: (1) In addition to obesity, there are physical diseases reported in the literature that can affect the gut microbiota, such as hypertension, diabetes, and digestive diseases; (2) in the last 6 months, drugs administration that might affect the gut microbiota, such as antibiotics, glucocorticoids, and high-dose probiotics; (3) medical examinations of the digestive tract, such as gastrointestinal, barium meal, etc., in the last 6 months; (4) surgery on the digestive tract and biliary tract in the last 5 years; (5) there were obvious changes in diet habits in the past 3 months; (6) there are obvious restrictions on movement due to physical diseases, such as bedridden.

### Clinical Evaluation

For the patients who met the inclusion and exclusion criteria, a questionnaire survey was conducted on all the subjects using a Case Report Form, including age, gender, ethnicity, occupation, height, weight, previous medical history, medication history, history of surgery, and consumptive history of tobacco and alcohol.

### Collection of Stool Samples

The patients were firstly instructed to urinate before defecation to prevent diluting or contaminating feces. The feces were drained into a clean container or on a clean urine pad. After defecation, the staff opened the sterile sampling bottle, peeled off the feces with a small spoon, which were on the inner cover of the sampling bottle, and dug the middle part of the feces. Repeatedly, dug feces into the sampling bottle until about 2-g samples were collected. The cap of the sampling bottle was then screwed tightly and quickly placed in a container containing liquid nitrogen for transportation. It was then transferred to a −80°C refrigerator and stored frozen within 1 h. The samples must be placed in a container filled with liquid nitrogen and attended by a special person no matter a short or long-distance transportation.

### 16S rRNA Amplification of the V3-V4 Region and Illumina Sequencing

According to the manufacturer’s instructions, a PowerSoil DNA kit (MoBio, United States) was used to extract 200-mg feces per sample for DNA extraction. The 16S rRNA (V3-V4) gene marker was amplified using KAPA HiFi HotStartReadyMix (KAPA, United States). Amplification was performed in triplicate by PCR. The amplicons were analyzed on a 1.5% agarose gel electrophoresis, and a band of a desired size was purified using a QIAquick gel extraction kit (QIAGEN, Hilden, Germany). The products were sequenced on the Illumina HiSeq 2500 platform and submitted to the second-generation sequencing laboratory of the Beijing Institute of Bioinformatics.

### Processing of Sequencing Data

Raw sequence data were processed and analyzed with QIIME software (Quantitative Analysis of Microbial Ecology, Version 1.9.1) (17). Fragments that contain ambiguous characters in the sequence or that contain more than two nucleotide mismatched primers need to be removed. When performing statistical analysis of biological information, to understand the number of bacteria and genus in a sample sequencing result, it was necessary to perform the classification operation and OTU division on all sequences according to the specified similarity (95, 97, or 98%, etc.). This study brought together sequences with at least 97% similarity, and used representative sequences from each cluster to identify bacterial taxa from the Greengenes database that was launched on 13 August 2013 (18).

### Statistical Analysis

Statistical analysis was performed using the R-3.3.1 and metagenomic data statistical analysis software (19). Visualization of the relationship between samples was performed with a principal coordinate analysis (PCoA) based on an unweighted UniFrac distance matrix, and significant differences in the composition of the microbial community were tested with ANOSIM (20). Samples were clustered using Jensen-Shannon distance and partitioning around medoid (PAM) clustering. Optimal number of clusters was estimated using Calinski-Harabasz (CH) index (12). We used the silhouette validation technique for assessing the robustness of the clusters. Participants whose gut microbiota mainly predominantly the abundace of Prevotella was major gut microbiota would be labeled as enterotype P, and the abundace of Bacteroides was major gut microbiota would be labeled as enterotype B. Based on this, the subjects could be divided into four groups: the patients with the enterotype-P group (SCH-P), the patients with the enterotype B group (SCH-B), the controls with the enterotype-P group (HC-P), and the controls with the enterotype B Group (HC-B).

Linear discriminant analysis (LDA) effect size (LEfSe, v1.0) was used to analyze the significant differences in relative abundance of gut microbiota categories related to the patients with the enterotype-P group and the controls with the enterotype-P group (21). Linear discriminant analysis (LDA) effect size (LEfSe) was used for the identification of the different markers, an alpha = 0.05 was used in Wilcoxon rank sum test, and the log value for the LDA analysis was set to be <2 (22). Moreover, the work further compared the differences in metabolic pathways of the patients with enterotype-P with the body mass index of more than 25 kg/m^2^ (the BMI-A group) and less than 25 kg/m^2^ (the BMI-N group). To obtain insight into the difference of the possible functional pathway between the two groups, we used PICRUSt to calculate contributions of various OTUs to evaluate the biological pathways based on KEGG orthology groups (KOs) using Kyoto Encyclopedia of Genes and Genomes (KEGG) databases (23).

Statistical analysis was performed using the SPSS19.0 software. Gender and tobacco and alcohol consumption of all the participants were expressed as a proportion or percentage, and the chi-square test was used for the count data. Independent *t*-test, Welch *t*-test, and White non-parametric *t*-test were used in continuous variables. For categorical variables between groups, the Pearson’s chi-square test or the Fisher’s exact test was used based on the validity of the hypothesis. The study took *p* < 0.05 as statistically significant.

## Results

### Demographic Characteristics

A total of 97 patients with schizophrenia and 69 healthy controls were eligible, and the male/female ratio of the patients and the controls groups was 43/54 and 33/36, respectively. The mean age of the patients was 46.93 ± 12.88 years and that of controls was 46.48 ± 12.24 years. There were no statistical differences between the two groups in terms of gender, age, smoking status, and drinking status (*p* > 0.05). There were 46 patients in the SCH-P, 51 patients in the SCH-B, 21 patients in the HC-P, and 48 patients in the HC-B.

### Distribution of BMI

**FIGURE 1:**
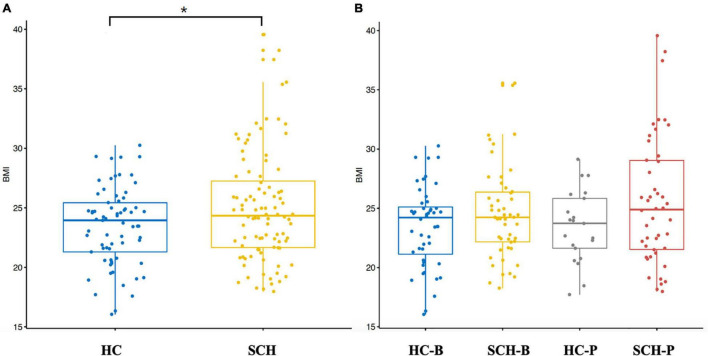
Box-plot **(A)** independent sample *t*-test was conducted for the two groups. BMI of schizophrenia was significantly higher than that of the healthy controls. **(B)** All the subjects were divided into four groups based on the enterotypes. Through analysis of variance, it was found that BMI of schizophrenic patients with Prevotella type showed an increasing trend. *p < 0.05.

By comparing the distribution of BMI, the BMI (25.26 ± 3.26 kg/m^2^) of the patients group was slightly higher than that of the controls group (24.12 ± 3.02 kg/m^2^), and there was a significant difference between the two groups (*p* < 0.05). On the basis of enterotype classification, the BMI of the SCH-P group (25.40 ± 3.27 kg/m^2^) had a tendency to increase compared with those of other groups (HC-B, 24.46 ± 3.28 kg/m^2^; SCH-B, 25.13 ± 3.28 kg/m^2^; HC-P, 23.32 ± 2.17 kg/m^2^) (Figure 1).

### Microbiota Differences Between the Patients and the Control Enterotype-P Group

**FIGURE 2:**
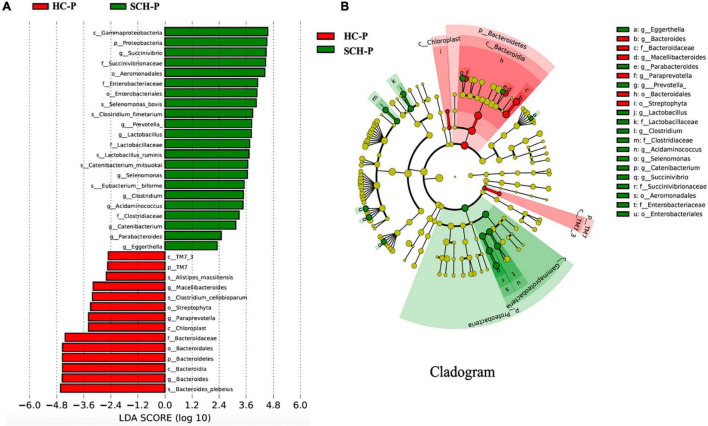
Differently abundant taxa identified using LEfSe analysis. **(A)** Visualization of only taxa meeting an LDA threshold >2. **(B)** LEfSe Cladogram showed the most differentially abundant taxa between the two groups. Taxa enriched for healthy with Prevotella-enterotype in red; schizophrenia with Prevotella-enterotype-enriched taxa in green. The brightness of each dot was proportional to its effect size.

By analyzing the gut microbiota difference and abundance of the patients and the controls, the abundance of Proteobacteria and Firmicutes in the SCH-P group was significantly higher than that in the HC-P group. Among them, the abundance of the Succinivibrio, Gammaproteobacteria, Proteobacteria, Succinivibrionaceae, Aeromonadales, and Enterobacteriaceae in the SCH-P group most significantly increased. In the HC-P group, the abundance of Bacteroides_plebeius, Bacteroides, Bacteroidia, Bacteroidetes, Bacteroidales, and Bacteroidaceae significantly increased (Figure 2).

### The Distribution Difference of Gut Microbiota of Enterotype-P in the Schizophrenia Group

**FIGURE 3:**
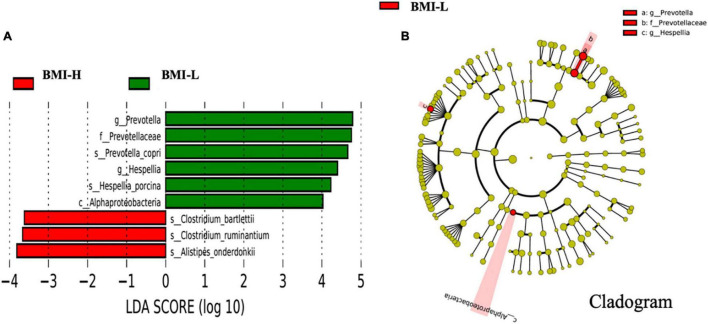
Differently abundant taxa identified using LEfSe analysis. **(A)** Visualization of only taxa meeting an LDA threshold >2. **(B)** LEfSe Cladogram showed the most differentially abundant taxa between the two groups. Taxa enriched for body mass index above 25 kg/m^2^ in red and below 25 kg/m^2^ in green. The brightness of each dot was proportional to its effect size.

The comparative analyses are performed within enterotype-P. According to body mass index, the subjects with schizophrenia enterotype-P were divided into the BMI normal group (BMI-H, 18–24.9 kg/m^2^) and the BMI abnormal group (BMI-L, ≥25 kg/m^2^). As shown in Figure 3, the abundance of Clostridium and Alistipes in the BMI-H group significantly increased, while the abundance of Prevotella, Hespellia, and Alphaproteobacteria in the BMI-L group dominantly increased (Figure 3).

**FIGURE 4:**
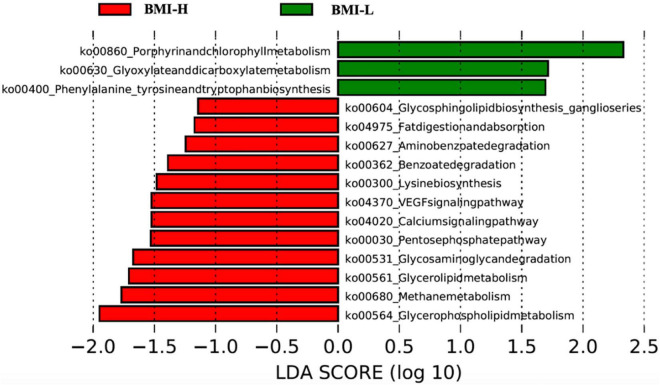
Pathway clustering analysis based on LefSe classification showed that gut microbiota in different weight ranges expressed different metabolic pathways. The gut microbiota of patients with high BMI can express more pathways related to disordered glucose and lipid metabolism.

According to clustering and differential analysis of metabolic pathways in the two groups by LEfSe analysis, there were several differences in the metabolic pathways of the microbiota with enterotype-P in different BMI intervals. The BMI-H group had outstanding effects on glycerolipid metabolism, glycerophospholipid metabolism, fat digestion and absorption, and degradation of glycosaminoglycans, thus affecting the body’s absorption and metabolism of fat (Figure 4).

## Discussion

This study verified that the proportion of obese patients in schizophrenia was higher than that in the normal population, which was consistent with the results of previous studies (24, 25). In addition, the gut microbiota of schizophrenia patients with obesity were alterations, which were characterized by the enterotype-P as the prominent. The enterotype was characterized by unique digestive functions and the preference for specific food substrates (26, 27). Among them, the enterotype-P is mainly classified by the Prevotella genus, which has abundant hydrolases that can specifically degrade plant fibers, but with the insufficient ability to decompose lipids and proteolytic fermentation. Majority of people with weaker lipid metabolism belong to this type of enterotype. A high-fiber diet may help enterotype-P patients to control their body weight (26, 27), which was contrary to the diet habits of patients with schizophrenia. Due to the abnormal metabolism of lipids in the intestine, the patients with schizophrenia were more prone to chylous diarrhea. In this study, the subjects with high BMI of enterotype-P showed relatively good lipid metabolism, which may be due to the environmental adaptation of the gut microbiota under the effect of high-fat and high-calorie diet in schizophrenia.

Compared with other enterotypes, enterotype-P fecal samples could ferment *in vitro* to produce more short-chain fatty acids, especially the levels of propionate were more prominent (28). Studies have shown that propionate could inhibit human feeding behaviors by regulating intestinal hormones (29). In the study of Chambers ES et al., propionate was found to significantly promote the release of intestinal hormones, such as peptide YY and glucagon-like peptide-1, in human colon cells (30). The increase of these substances promoted the reduction of energy intake, and long-term supplementation of propionate could effectively reduce weight gain, distribution of adipose tissue in the abdominal cavity, and lipid content in liver cells, and prevent deterioration of insulin sensitivity. Those activities may all improve the status of obesity (31, 32). In addition, *in vitro* studies have shown that propionic acid can regulate mitochondrial function and various biochemical processes related. Propionic acid can partially reverse the decline in mitochondrial function caused by oxidative stress (33). Therefore, enterotype changes in patients with schizophrenia may play a key role for the gut microbiota in the human body to reverse the situation of obesity.

## Conclusion

In conclusion, this study found that the patients with schizophrenia had a significantly higher BMI than that of the health controls. The patients with enterotype-P had a higher BMI. The enterotype-P might affect a variety of metabolic pathways to disturb the metabolism of glucose and lipid in human body.

## Data Availability Statement

The datasets presented in this study can be found in online repositories. The names of the repository/repositories and accession number(s) can be found below: CNCB National Genomics Data Center, accession no: CRA000653.

## Ethics Statement

The studies involving human participants were reviewed and approved by the Ethics Committee of Kangning Hospital in Liaoning Province. The patients/participants provided their written informed consent to participate in this study.

## Author Contributions

YL designed the study and wrote the protocol. JG and YY managed the literature searches and analyses. MZ undertook the statistical analysis. YS and JG wrote the first draft of the manuscript. All authors contributed to the article and have approved the final manuscript.

## Conflict of Interest

YY and MZ was employed by the company Beijing Gene Tangram Technology Co., Ltd., Beijing, China. The remaining authors declare that the research was conducted in the absence of any commercial or financial relationships that could be construed as a potential conflict of interest.

## Publisher’s Note

All claims expressed in this article are solely those of the authors and do not necessarily represent those of their affiliated organizations, or those of the publisher, the editors and the reviewers. Any product that may be evaluated in this article, or claim that may be made by its manufacturer, is not guaranteed or endorsed by the publisher.
